# Speciation of arsenic trioxide metabolites in peripheral blood and bone marrow from an acute promyelocytic leukemia patient

**DOI:** 10.1186/1756-8722-5-1

**Published:** 2012-01-24

**Authors:** Noriyoshi Iriyama, Yuta Yoshino, Bo Yuan, Akira Horikoshi, Yukio Hirabayashi, Yoshihiro Hatta, Hiroo Toyoda, Jin Takeuchi

**Affiliations:** 1 Department of Hematology and Rheumatology, Nihon University School of Medicine, Itabashi Hospital, Tokyo, Japan; 2 Department of Clinical Molecular Genetics, School of Pharmacy, Tokyo University of Pharmacy & Life Sciences, Tokyo, Japan; 3 Department of Internal Medicine, Nihon University School of Medicine, Nerima-Hikarigaoka Hospital, Tokyo, Japan

**Keywords:** Acute promyelocytic leukemia, Arsenic trioxide, Arsenic metabolite, Bone marrow, High-performance liquid chromatography/inductively coupled plasma mass spectrometry, Arsenic speciation

## Abstract

**Background:**

Speciation of arsenic trioxide (ATO) metabolites in clinical samples such as peripheral blood (PB) from acute promyelocytic leukemia (APL) patients has been conducted. However, speciation of arsenicals in bone marrow (BM) has not yet been performed. Profiles of arsenic speciation in plasma of BM were thus investigated and compared with those of PB plasma from a relapsed APL patient. The total arsenic concentrations in high molecular weight fraction (HMW-F) of BM and PB plasma were also determined.

**Methods:**

Response assessment was evaluated by BM aspirate examination and fluorescence in situ hybridization analysis. The analyses of total arsenic concentrations and speciation were preformed by inductively coupled plasma mass spectrometry (ICP-MS), and high-performance liquid chromatography (HPLC)/ICP-MS, respectively.

**Results:**

Response assessment showed that the patient achieved complete remission. The total arsenic concentrations in BM plasma increased with time during the consecutive administration. The PB plasma concentrations of methylated arsenic metabolites substantially increased after the start of administration, while those of inorganic arsenic were still kept at a low level, followed by substantially increase from day-14 after administration. The arsenic speciation profiles of PB plasma were very similar to those of BM plasma. Furthermore, the total arsenic concentrations of HMW-F in BM plasma were much higher than those in PB plasma.

**Conclusions:**

The behaviors of arsenic speciation suggested for the first time that arsenic speciation analysis of PB plasma could be predicative for BM speciation, and showed relatively higher efficiency of drug metabolism in the patient. These results may further provide not only significance of clinical application of ATO, but also a new insight into host defense mechanisms in APL patients undergoing ATO treatment, since HMW proteins-bound arsenic complex could be thought to protect BM from the attack of free arsenic species.

## Introduction

Acute promyelocytic leukemia (APL) is a unique subtype of acute myeloid leukemia (AML) and accounts for approximately 10-15% of all cases of AML in adults [1]. It is also characterized by a specific cytogenetic reciprocal chromosome translocations, t(15;17), generating PML/RARα fusion gene, which is thought to play a central role in the initiation of leukemogenesis [2-5]. An introduction of all*-trans *retinoic acid (ATRA) since 1986 has dramatically improved the outcome of treatment of this disease [3]. Nevertheless, an approximately 30% of the patients relapse and often become resistant to the conventional treatment with ATRA alone, or in combination with chemotherapy [5]. On the other hand, investigators from China and the USA have demonstrated that the treatment with arsenic trioxide (ATO, As(III)) could induce complete remission (CR) in 90% of relapsed patients [1,6,7].

We have been investigating the effects of As(III) using a unique in vitro system comprising primary cultured chorion and amnion cells prepared from human fetal membranes [8-10], and demonstrated that aquaporin 9 and multidrug resistance-associated protein 2 are functionally involved in controlling arsenic accumulation in these normal cells, which then contribute to differential sensitivity to As(III) cytotoxicity between these cells [11]. These findings may have broad important implications for revealing the mechanisms underlying the side effects of ATO in patients. Furthermore, in order to understand the mode of actions of ATO and provide an effective treatment protocol for individual APL patients, studies have been conducted on the pharmacokinetics of ATO in APL patient using biological samples such as urine, blood and cerebrospinal fluid [12-14]. In fact, we recently demonstrated that various arsenic species including inorganic arsenic and methylated arsenic metabolites accumulated in red blood cells (RBCs) in an APL patient [13]. We have also demonstrated for the first time that these arsenic metabolites also existed in cerebrospinal fluid [14], in which the total arsenic concentrations reached levels necessary for differentiation induction [1,15]. These findings may provide a new insight into clinical applications of ATO, and better therapeutic protocols [16].

It has become clear that like hematopoietic stem cells, leukemia stem cells (LSCs), which acquired limitless self-renewal through oncogenic transformation, also reside in bone marrow (BM) microenvironment, and contribute to maintain the acute myeloid leukemia phenotype [17]. Recent studies have also demonstrated that incomplete eradication of primary LSCs is closely linked to chemotherapy resistance, and consequently contribute to eventual disease relapse [17,18]. In this regard, it is significant to note that targeting for the PML with ATO could lead to loss of self-renewal capability of LSCs in chronic myeloid leukemia (CML) based on the studies on stem cells isolated from BM of human CML patients and mouse bone marrow transplantation models [18]. These findings suggest that ATO would be a promising candidate for a LSCs-targeted therapy and raise a concern as to the detailed distribution profiles of arsenic in BM. These ideas are based on previous findings indicating that the total arsenic concentrations in the plasma of BM from five relapsed APL patients were close to levels for inducing differentiation, yet the analysis was conducted for the BM sample collected at just only one collection time point [19]. Furthermore, to the best of our knowledge, no speciation analysis of arsenicals in BM from APL patients undergoing long-term administration of ATO has been done before, despite the fact that BM is a vital site for regulating the production of blood cells.

It has been established that biomethylation is a major metabolic pathway for inorganic arsenic in human beings as well as many animal species, in which As(III) is methylated to form trivalent and pentavalent products such as methylarsonous acid (MA(III)), methylarsonic acid (MA(V)), dimethylarsinous acid (DMA(III)), dimethylarsinic acid (DMA(V)) [16]. Furthermore, it has become clear that trivalent arsenicals can interact with biological molecules of importance containing sulfhydryls [20]. In fact, a study on complexes of trivalent arsenicals with proteins has been conducted using organ samples such as livers and kidneys in rats after an intravenous injection of arsenite [21]. However, no such studies have been conducted in biological samples from APL patients undergoing ATO treatment. Furthermore, the biological as well as clinical importance of proteins-bound arsenic complex has been proposed [20,22], yet the detailed studies have not been preformed.

In this study, we evaluated the clinical efficiency of ATO in a relapsed APL patient undergoing long-term administration of ATO by response assessment. In order to obtain a general view regarding the pharmacokinetic behaviors of ATO in APL patients, we also performed a detailed systematic analysis of the accumulation of ATO in RBCs as well as its metabolites in plasma of peripheral blood (PB). Most importantly, besides total arsenic determination, we further investigated for the first time the arsenic speciation in plasma of BM, and compared the arsenic speciation profiles between PB and BM in order to gain more detailed information on the distribution of arsenic. Furthermore, the total arsenic concentrations of high molecular weight fraction (HMW-F) in BM and PB plasma were determined. Additionally, we collected clinical samples just before (within 30 minutes) the start of daily administration in order to get accurate blood trough levels, since the levels are well known to be closely related to clinical outcomes.

## Patient and methods

### Patient characterization

A 49-year-old Japanese woman was diagnosed with APL in September 2003, and achieved first CR with ATRA. CR was kept up to 15 months by the treatment with consolidation chemotherapy according to JALSG APL97 regimen [23]. However, she relapsed in April 2005. After receiving reinduction therapy with ATRA, second CR was obtained, followed by two 7-day course of consolidation chemotherapy comprising mitoxantrone 6 mg/m^2 ^intravenous (IV) bolus from day 5 to 7, etoposide 100 mg/m^2 ^IV administration for 1 h from day 1 to day 5, and cytarabine (Ara-C) 150 mg/m^2 ^continuous IV infusion for 24 h from day 1 to day 6 (MEC therapy). However, the second relapse was confirmed in November 2009.

### Treatment protocol

The complete treatment protocol was approved by the Internal Review Committee of Nihon University, and a written informed consent was obtained from the study patient before the treatment start. ATO monotherapy was administered daily intravenously for 2 h at a dose of 0.15 mg/kg for 43 consecutive days. No critical complications such as disseminated intravascular coagulation (DIC), organ failure, bleeding tendency, infectious disease, APL differentiation syndrome, or QT prolongation on electrocardiogram were observed during the administration period.

To evaluate the pharmacokinetics of arsenic species precisely, we controlled the daily diet, in particular seafood, during the periods of remission induction therapy, since arsenic compounds such as arsenobetaine (AB) exist in seafood, and can make analysis complex during remission induction and/or consolidation therapy [13].

### Response assessment

Bone marrow aspirations for morphology and cytogenetics examination were performed by conventional methods at Nihon University School of Medicine, Itabashi Hospital, Tokyo, Japan. For morphologic response, a clinical CR was defined as a bone marrow aspirate with ≤ 5% blast cells plus promyelocytes with no evidence of leukemic cells [24,25]. Furthermore, flow cytometry analysis for cell surface markers of the blastoid fraction by assessing the expression levels of CD11b and CD15 associated with myeloid maturation, and CD117 and CD34 for primitive myeloid cells, were conducted at SRL (Special Reference Laboratories, Tokyo, Japan). Conventional cytogenetic test for t(15:17) was performed by fluorescence in situ hybridization (FISH) analysis and a negative FISH result was considered as a cytogenetic complete remission.

### Preparations of clinical samples

Blood and bone marrow samples were collected in an EDTA-containing collection tube during the remission induction therapy. Blood samples were collected before treatment (day -1), and 3, 7, 10, 14, 17, 21, 28, 42, and 56 days after the start of administration. Bone marrow aspirations were performed before treatment (day -1), and 14, 28, 42, and 56 days after the start of administration. Both blood and bone marrow samples were collected just before (within 30 minutes) the start of daily administration, and the concentrations of arsenic at this time-point were considered as its blood trough level during the consecutive administration. Samples were separated immediately into red blood cells and plasma by centrifugation at 1,000 g for 10 min at 4°C. Subsequently, the RBCs and plasma were stored at -35°C until analysis.

### Sample preparations for arsenic analysis

Samples for total arsenic determination and arsenic speciation were prepared as described previously [13]. Since it is difficult to measure volume of plasma and blood cells correctly using a Pipetman due to a viscous nature of samples, we measured weight of samples in each experiment. Briefly, RBCs and plasma (0.2 g each) were taken into a 15 mL polypropylene centrifuge tube. The samples were mixed with HNO_3 _(0.5 mL) at room temperature for 10 min, and incubated at 80-110°C on hot plate after addition of 30% H_2_O_2 _(0.25 mL). They were then diluted with Milli-Q water to 5 mL and filtrated with 0.45 μm membrane filter (Millex-HA, MILLIPORE, USA). Filtrates were subjected to total arsenic determination. On the other hand, in order to prepare samples for arsenic speciation, the plasma (0.2 g) was ultrafiltrated with a 10-kDa molecular mass cutoff (Micrcon centrifugal filter devices 10,000 MWCO, Millipore, USA). The filtrates were thus defined as low molecular weight fraction (LMW-F) and subjected to arsenic speciation analysis. The remains trapped in filters were defined as high molecular weight fraction (HMW-F) and subjected to total arsenic determination.

### Analysis of total arsenic concentrations and arsenic speciation

The analysis of total arsenic concentrations and arsenic speciation were performed by inductively coupled plasma mass spectrometry (ICP-MS) (ELAN DRC-e, PerkinElmer SCIEX, ON, Canada), and high-performance liquid chromatography (HPLC) (LC PU611 VS, GL Science, Tokyo, Japan)/ICP-MS, respectively, as described previously [13]. CAPCELL PAC C18 MG II (4.6 mm I.D. × 250 mm, Shiseido Inc., Tokyo, Japan) was used as the separation column on HPLC system, and MG II cartridge was attached as a guard column to allow direct injection of biological samples. The previously reported mobile phase conditions for HPLC by our group [13] were used in the current study. The retention times were determined with TotalChrom Workstation version 6.2.0 (PerkinElmer SCIEX, ON, Canada). The quantitation of arsenic concentrations was performed by external calibration. Concentrations of arsenic compounds were calculated from a calibration curve of standard arsenic compounds. The peak area was determined by TotalChrom Workstation version 6.2.0. As the standard arsenic compounds, sodium arsenate (As(V)), sodium arsenite (As(III)), methylarsonic acid (MA(V)), dimethylarsinic acid (DMA(V)) and arsenobetaine (AB) were purchased from Tri Chemical Laboratories Inc. (Yamanashi, Japan).

## Results

### Treatment efficacy

As shown in Table 1, the examination of bone marrow aspirations showed that the percentage of blast cells plus promyelocytes was 0.8% on day 28, and was kept less than 5% until day 56, although a transient increase was observed on day 14, indicating that the clinical CR was achieved on day 28. Furthermore, PML/RARa fusion gene was not detected on day 56 by FISH testing, indicating that the patient achieved cytogenetic CR after remission induction therapy with ATO. Additionally, differentiation induction of APL cells was confirmed by a substantial increase in the expression levels of CD11b and CD15, accompanying with a substantial decrease in the expression levels of CD117 and CD34 throughout the remission induction therapy. Examination of bone marrow also showed nuclear cell count of 4.0 to 12.0 × 10,000/μL containing 21.6 to 51.2% of myeloid; 22.6 to 70.0% of erythroid; and 7.2 to 32.0% of others. Furthermore, peripheral blood test results showed white blood cell count ranging from 1.0 to 2.3 × 1,000/μL; neutrophil cell count ranging from 0.15 to 1.1 × 1,000/μL; hemoglobin concentration ranging from 10.9 to 13.6 g/dL; platelet count ranging from 11.5 to 18.0 × 10,000/μL. During the remission induction therapy, there was no requirement of any additional treatments for complications or blood transfusion.

**Table 1:** Response assessment during the remission induction therapy

Days after the start of administration	-1	14	28	42	56
Nuclear cell count (× 10^4^/μL)	6.0	7.0	10.0	12.0	4.0
Blast+Promyelocyte (%)	5.8	17.8	0.8	1.2	0.6
Myeloid (%)	51.2	32.6	29.4	21.6	34.2
Erythroid (%)	22.6	28.8	55.0	70.0	33.2
Others (%)	20.4	20.8	14.8	7.2	32.0
PML-RARα FISH (%)	5.0	83.0	33.0	9.0	0.0
CD11b (%)	5.0	27.0	43.2	59.3	ND
CD15 (%)	8.6	23.2	38.5	47.2	ND
CD117 (%)	94.5	30.4	41.3	23.9	ND
CD34 (%)	38.0	14.4	26.2	15.8	ND

BM aspirations were collected before the treatment start (day -1), and 14, 28, 42, and 56 days after the start of administration. Response assessment was evaluated by BM aspirate examination, FISH analysis and the expression levels of cell surface markers. ND: no data.

### Total arsenic concentrations in PB RBCs, PB plasma and BM plasma

**Figure 1:**
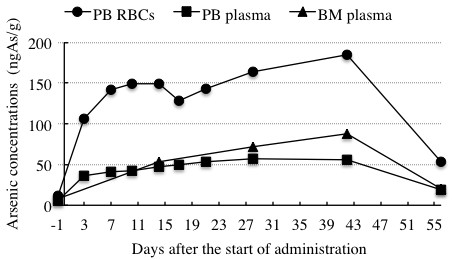
**Profiles of total arsenic concentrations in PB RBCs, PB plasma and BM plasma collected during the remission induction therapy**. Symbols (●), (■) and (▲) show the total arsenic concentrations in PB RBCs, PB plasma and BM plasma, respectively. PB, peripheral blood; RBCs, red blood cells; BM, bone marrow.

Profiles of total arsenic concentrations in the PB RBCs, PB plasma and BM plasma are shown in Figure 1 and summarized in Table 2. Total arsenic concentrations in the PB RBCs, and PB plasma ranged from 11.6 ngAs/g (day -1) to 184.8 ngAs/g (day 42), and from 5.55 ngAs/g (day -1) to 55.5 ngAs/g (day 42), respectively, indicating that total arsenic concentrations in RBCs and plasma of PB increased with time during the consecutive administration. Furthermore, total arsenic concentrations in the PB RBCs were approximate 3 times higher than those in PB plasma during the remission induction therapy. At the same time period, total arsenic concentrations in the BM plasma ranged from 7.59 ngAs/g (day -1) to 88.1 ngAs/g (day 42), indicating that arsenic accumulation was observed in not only PB RBCs and PB plasma but also BM plasma. Moreover, total arsenic concentrations tended to be higher in the BM plasma than those in the PB plasma. On the other hand, total arsenic concentrations in these biological samples started decreasing markedly from the last administration (day 42).

**Table 2:** Total arsenic concentrations in PB RBCs, PB plasma and BM plasma collected during the remission induction therapy

Days after the start of administration		-1	3	7	10	14	17	21	28	42	56
Arsenic concentrations (ngAs/g)	PB RBCs	11.6	106.2	141.4	149.7	149.4	128.2	142.7	163.6	184.8	53.7
	PB Plasma	5.55	36.6	41.1	42.2	47.6	49.7	53.0	56.6	55.5	18.5
	BM Plasma	7.59	ND	ND	ND	53.5	ND	ND	72.1	88.1	20.4

Blood samples were collected before the treatment start (day -1), and 3, 7, 10, 14, 17, 21, 28, 42, 56 days after the start of administration. The total arsenic concentrations in each clinical sample were determined by ICP-MS as described in "Patient and Methods". ND: no data.

### Arsenic speciation in PB plasma

**Figure 2:**
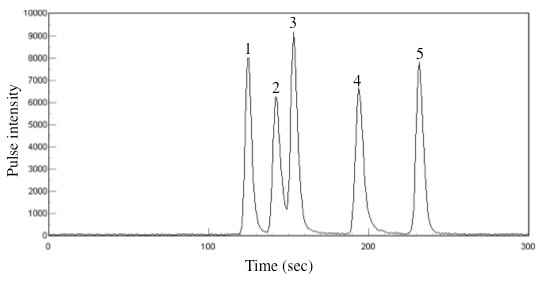
**Chromatogram of standard arsenic species**. The separation was performed on CAPCELL PAC C18 MG II with 10 mM butane sulfonic sodium, 4 mM malonic acid, 4 mM tetramethylammonium hydroxide, and 0.5% methanol (pH 2.0). The signal peaks are as follows: As(V) (1), As(III) (2), MA(V) (3), DMA(V) (4), and AB (5).

**Figure 3:**
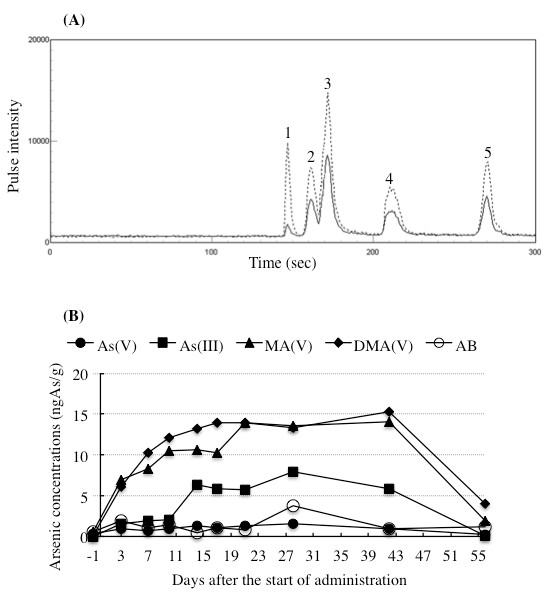
**Profiles of concentrations of arsenic species in PB plasma collected during the remission induction therapy**. Blood samples were collected before the treatment start (day -1), and 3, 7, 10, 14, 17, 21, 28, 42, 56 days after the start of administration. The concentrations of arsenic species in plasma were determined with HPLC/ICP-MS as described in "Patient and Methods." A merged representative chromatogram obtained from the analyses of a plasma sample collected on day 14 and the same plasma sample spiked with As(V), As(III), MA(V), DMA(V), and AB is shown in Figure 3(A). The signal peaks are as follows: As(V) (1), As(III) (2), MA(V) (3), DMA(V) (4), and AB (5). The concentrations of arsenic species calculated from these chromatograms are shown in Figure 3(B). (●): As(V); (■): As(III); (▲): MA(V); (♦): DMA(V); (O): AB.

A representative chromatogram of standard arsenic compounds demonstrated the following elution order of As(V), As(III), MA(V), DMA(V) and AB (Figure 2). A representative merged chromatogram obtained from the analyses of a plasma sample collected on day 14 and the same plasma sample spiked with As(V), As(III), MA(V), DMA(V), and AB is shown in Figure 3(A). Coelution of the spiked arsenic standard with the suspected compound in the plasma demonstrated their same chromatographic behavior (Figure 3(A)). Furthermore, coelution of the spiked arsenic standard with the suspected compound in other plasma also demonstrated the same chromatographic behavior (data not shown).

Profiles of As(V), As(III), MA(V), DMA(V) and AB are shown in Figure 3(B) and summarized in Table 3. Before the start of administration (day -1), no As(III) and only a small amount of As(V), MA(V), and DMA(V) were detected. However, after the start of administration, the concentrations of MA(V) and DMA(V) substantially increased with time and almost reached a plateau from day 21. Moreover, the concentrations of DMA(V) tended to be higher than MA(V), especially in the initial stage of administration (day 7-17), and even after the last administration (from day 42). It should be noted that As(III) concentrations substantially increased from day 14 and the increase continued up to day 42, although an apparent increase in the concentrations was observed during the initial treatment period (from day 3 to day 10). Regarding As(V), the concentrations increased slightly during the remission induction therapy compared with those of other arsenic species. Furthermore, the concentrations of DMA(V) and MA(V) were still higher than those of other arsenic metabolites on day 56, which is the day 13 after the last administration. At the same time, the concentrations of AB increased slightly and were maintained at almost the same level except day 28 when compared with those of the base line (day -1), which could be a result of seafood intake at restaurant during the remission induction therapy.

**Table 3:** Arsenic concentrations in PB plasma collected during the remission induction therapy

Days after the start of administration		-1	3	7	10	14	17	21	28	42	56
Arsenic concentrations (ngAs/g)	As(V)	0.36	0.96	0.69	0.99	1.26	1.03	1.34	1.50	0.99	0.24
	As(III)	0	1.50	1.86	2.02	6.31	5.89	5.74	7.89	5.83	0.09
	MA(V)	0.55	6.91	8.30	10.46	10.59	10.24	13.99	13.50	14.05	1.87
	DMA(V)	0.11	6.14	10.3	12.2	13.2	14.0	14.0	13.3	15.3	3.96
	AB	0.60	1.94	1.06	1.39	0.43	1.04	0.88	3.80	0.99	1.22

Blood samples were collected before the treatment start (day -1), and 3, 7, 10, 14, 17, 21, 28, 42, 56 days after the start of administration. The concentrations of arsenic species in PB plasma were determined by HPLC/ICP-MS as described in "Patient and Methods".

### Arsenic speciation in BM plasma

**Figure 4:**
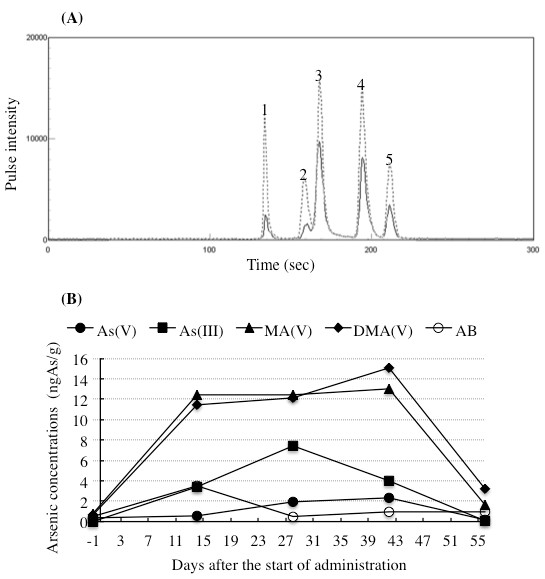
**Profiles of concentrations of arsenic species in BM plasma collected during the remission induction therapy**. BM aspirates were collected before the treatment start (day -1), and 14, 28, 42, and 56 days after the start of administration. The concentrations of arsenic species in plasma were determined with HPLC/ICP-MS as described in "Patient and Methods." Similar to Figure 3 (A), identification of arsenic species by a spike test was conducted using a plasma sample collected on day 14 and was shown in Figure 4 (A). The concentrations of arsenic species calculated from these chromatograms are shown in Figure 4 (B). (●): As(V); (■): As(III); (▲): MA(V); (♦): DMA(V); (O): AB.

Similar to arsenic speciation in PB plasma, identification of arsenic species by a spike test was also conducted using a BM plasma sample collected on day 14. Coelution of the spiked arsenic standard with the suspected compound in the plasma demonstrated their same chromatographic behavior (Figure 4(A)). Furthermore, coelution of the spiked arsenic standard with the suspected compound in other plasma also demonstrated the same chromatographic behavior (data not shown).

Profiles of As(V), As(III), MA(V), DMA(V) and AB are shown in Figure 4(B) and summarized in Table 4. Just like in Figure 3(B) and Table 3, no As(III) and only a small amount of As(V), MA(V), and DMA(V) were detected on the day (day -1) before the start of administration. However, the concentrations of MA(V) and DMA(V) remarkably increased from day 14 and the increase continued up to day 42, a profile of which is similar to that observed in PB plasma. Moreover, the concentrations of DMA(V) tended to be higher than those of MA(V) in later stage of administration (from day 28). Additionally, the concentrations of As(III) substantially increased from day 14 and reached its peak on day 28, followed by declining over time. Similar to the profiles of As(V) in PB plasma, its concentrations increased slightly compared with those of other arsenic species. Again, DMA(V) and MA(V) were still primary metabolites in BM plasma as observed in PB plasma. As expected, the alterations of AB concentrations caused by patient's seafood intake were also observed in BM plasma.

**Table 4:** Arsenic concentrations in BM plasma collected during the remission induction therapy

Days after the start of administration		-1	14	28	42	56
Arsenic concentrations (ngAs/g)	As(V)	0.35	0.54	1.91	2.34	0.17
	As(III)	0	3.43	7.47	4.00	0.10
	MA(V)	0.80	12.4	12.4	13.0	1.63
	DMA(V)	0.63	11.5	12.1	15.1	3.21
	AB	0.51	3.49	0.50	0.99	1.00

BM aspirates were collected before the treatment start (day -1), and 14, 28, 42, and 56 days after the start of administration. The concentrations of arsenic species in BM plasma were determined by HPLC/ICP-MS as described in "Patient and Methods".

### Comparison of arsenic speciation profiles between PB plasma and BM plasma

**Figure 5:**
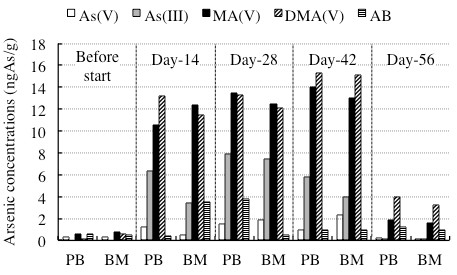
**Comparison of arsenic speciation profiles between PB plasma and BM plasma**. The columns of Before start, Day-14, Day-28, Day-42 and Day-56 represent the speciation profiles of PB and BM plasma collected before the treatment start (day -1), and 14, 28, 42, and 56 days after the start of administration, respectively. The open column, gray column, black column, hatched column and vertical striped column show As(V), As(III), MA(V), DMA(V) and AB, respectively.

In order to clarify whether there is a similar arsenic speciation profiles in PB and BM plasma, we combined arsenic speciation analysis data of PB and BM plasma obtained at the same time point and summarized in Figure 5 and Table 5. Although a slight difference in the concentrations of respective arsenic metabolites was observed at each time point, the arsenic speciation pattern between PB and BM plasma showed a close similarity throughout the remission induction therapy.

**Table 5:** Arsenic concentrations in PB and BM plasma collected during the remission induction therapy

Days after the start of administration		-1		14		28		42		56	
Clinical samples		PB	BM	PB	BM	PB	BM	PB	BM	PB	BM
Arseni concentrations (ngAs/g)	As(V)	0.36	0.35	1.26	0.54	1.50	1.91	0.99	2.34	0.24	0.17
	As(III)	0	0	6.31	3.43	7.89	7.47	5.83	4.00	0.09	0.10
	MA(V)	0.55	0.80	10.6	12.4	13.5	12.4	14.0	13.0	1.87	1.63
	DMA(V)	0.11	0.63	13.2	11.5	13.3	12.1	15.3	15.1	3.96	3.21
	AB	0.60	0.51	0.43	3.49	3.80	0.50	0.99	0.99	1.22	1.00

Blood samples and BM aspirations were collected before the treatment start (day -1), and 14, 28, 42, and 56 days after the start of administration. The concentrations of arsenic species in PB and BM plasma were determined by HPLC/ICP-MS as described in "Patient and Methods".

### Total arsenic concentrations in high molecular weight fraction (HMW-F) of PB and BM plasma

**Figure 6:**
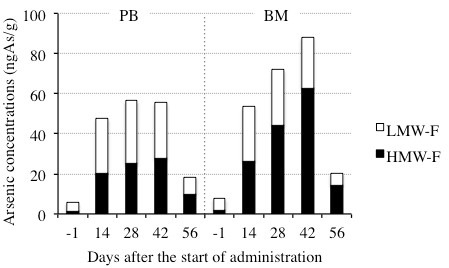
**Total arsenic concentrations in HMW-F and LMW-F of PB and BM plasma**. Blood samples and BM aspirations were collected before the treatment start (day -1), and 14, 28, 42, and 56 days after the start of administration. HMW-F of PB and BM plasma were prepared and subjected to total arsenic determination as described in "Patient and Methods". The total arsenic concentrations in LMW-F of PB and BM plasma were obtained by subtraction of that in HMW-F from the total arsenic concentrations in PB and BM plasma.

We also determined total arsenic concentrations of HMW-F in PB and BM plasma to obtain more detailed profiles of arsenic distribution. The total arsenic concentrations of HMW-F were much higher in BM plasma than those in PB plasma (Figure 6 and Table 6), indicating that the underlying reason for a higher total arsenic concentration in BM plasma than in PB plasma as shown in Figure 1 is attributed to the difference in the amount of arsenic contained in HMW-F, since the amount of arsenic in LMW-F was almost the same.

**Table 6:** Total arsenic concentrations in HMW-F and LMW-F of PB and BM plasma collected during the remission induction therapy

Clinical samples		PB					BM				
**Days after the start of administration**		**-1**	**14**	**28**	**42**	**56**	**-1**	**14**	**28**	**42**	**56**

Arsenic concentrations (ngAs/g)	Total plasma	5.55	47.6	56.6	55.5	18.5	7.59	53.5	72.1	88.1	20.4
	HMW-F of plasma	1.31	20.1	25.0	27.7	9.98	1.77	26.3	44.2	62.9	14.4
	LMW-F of plasma	4.24	27.5	31.5	27.8	8.54	5.83	27.3	27.9	25.2	5.99

PB and BM aspirations were collected before the treatment start (day -1), and 14, 28, 42, and 56 days after the start of administration. The total arsenic concentrations of HMW-F of PB and BM were determined by ICP-MS as described in "Patient and Methods". The total arsenic concentrations in LMW-F of PB and BM plasma were obtained by subtraction of that in HMW-F from the total arsenic concentrations in PB and BM plasma.

## Discussion

Administration of ATO has demonstrated a remarkable efficacy in the treatment of relapsed and refractory APL patients, who are generally resistant to the combinatory conventional treatment protocol consisting of ATRA and chemotherapy [1,2,6,7,16,26]. Consistent with these previous reports, the present study reconfirmed the clinical efficacy of ATO in a relapsed APL patient who achieved CR with ATRA once. Similar to a previous review report in which the median time to achieve CR in patients treated with ATO alone ranged from 28 to 38 days [27], the patient in the present study also achieved CR on day 28 (Table 1). We also demonstrated a substantial induction of APL cells differentiation in the patient after the treatment with ATO as evidenced by the alterations of the expression levels of cell surface antigens associated with myeloid maturation (Table 1). Results of the differentiation induction are in good agreement with other experimental results showing that a relatively lower dose of ATO induces differentiation of freshly isolated APL cells from patients as well as NB4 cells [1,2,15,16]. Of note, there was no requirement of any additional treatment for complications or blood transfusion and no drug discontinuation throughout the remission induction therapy, although some clinical data failed to return to a normal clinical reference range probably due to immediate determination after treatment with ATO. These results suggested that the patient would be a good model for studying the pharmacokinetics as well as the pharmacodynamics of ATO.

In agreement with our previous report [13], our present results clearly demonstrated that the total arsenic concentrations in either PB RBCs or PB plasma increased with time during the consecutive administration, and that the majority of arsenic was present in the RBCs (Figure 1 and Table 2). Therefore, careful attention should be paid to profiles of arsenic species in RBCs. We also demonstrated for the first time a time-dependent increase of the total arsenic concentrations in BM plasma, which was similar to that in PB plasma (Figure 1 and Table 2). Furthermore, the total arsenic concentration levels tended to be higher in the BM plasma than those in the PB plasma, raising clinical concerns and inspiring us to unravel the detailed information on the distribution of arsenic as well as its speciation in these biological samples.

We have previously demonstrated that the PB plasma concentrations of both methylated arsenic metabolites (MA(V) and DMA(V)), and inorganic arsenic (As(V) and As(III)) remarkably increased after the start of administration in a Japanese APL patient undergoing consolidation therapy [13]. Intriguingly, in the current study, the concentrations of MA(V) and DMA(V) increased substantially after the start of administration, while those of As(III) were still kept at a low level until day 10, followed by substantial increase from day 14 (Figure 3(B) and Table 3). It has been reported that in many animal species including human beings, biomethylation is a major metabolic pathway for inorganic arsenic, through which arsenic undergoes metabolic conversion by the reduction of As(V) to As(III) with subsequent methylation, yielding MA(V) then to DMA(V) [16,28,29]. Compared to the APL patient in our previous study [13], the patient enrolled in the current study appeared to have relatively higher metabolic efficiency probably due to her relatively young age or without clinical complications. Results from previous studies and our present study suggest that the efficiency of drug metabolism is obviously different in individual patients with different backgrounds, such as age range, with or without organ failure or DIC, which in turn affect clinical outcomes and appearance of side effects.

Of note, a previous study on the pharmacokinetics of ATO in Japanese patients with relapsed or refractory APL [30] demonstrated that the PB plasma concentrations of inorganic arsenic reached the steady state during the consecutive administration, while the methylated arsenic metabolites increased in relation to administration frequency. On the other hand, our recent study involving an APL patient with first relapse demonstrated that the PB plasma concentrations of both inorganic arsenic and methylated arsenic metabolites increased with administration frequency [13]. Interestingly, a trend toward reaching a plateau in these arsenic species was observed in PB plasma in the current study (Figure 3(B) and Table 3). These results clearly suggest that arsenic metabolism among individual patients were different. A similar observation was reported by Wang et al. [12], in which there are interindividual differences in excretion profiles and the relative concentrations of major arsenic species in urine among four Chinese APL patients undergoing ATO treatment. It is significant to note that genetic polymorphisms in arsenic metabolism genes, such as human arsenic methyltransferase (AS3MT) gene and glutathione S-transferase gene, are considered to be related to interindividual variation in the arsenic metabolism [31-33]. Collectively, continuous efforts to understand the differences in arsenic metabolism and the relationship between these genetic polymorphisms and arsenic metabolism among patients are definitely useful for providing an effective treatment protocol of ATO for individual APL patients, ultimately contribute to reduction of its side effects. Moreover, it should be noted that a close similarity of the arsenic speciation profiles between PB and BM plasma was observed throughout the remission induction therapy (Figure 5 and Table 5). These results thus suggested for the first time that arsenic speciation analysis of PB plasma could be predicative for BM speciation without applying BM aspiration.

We then focused on the total amount of arsenic in HMW-F trapped in a 10-kDa molecular mass cutoff filters and demonstrated that its concentrations were much higher in BM plasma than those in PB plasma (Figure 6 and Table 6). One important biological effects of arsenic has been suggested to be mediated by reaction with closely spaced cysteine residues on critical cell protein [34]. Several proteins such as tubulin, thioredoxin reductase, AS3MT with a high cysteine content and accessible thiol group are candidates for interactions with arsenic [16,20]. In fact, arsenic bound to high molecular weight proteins (MW > 10-kDa) has been detected in livers and kidneys in rats after an intravenous injection of arsenite [21]. Based on the vital role of BM microenvironment in maintaining the homeostasis of hematopoietic system, we assumed that a higher amount of proteins (MW > 10-kDa)-bound arsenic complex contribute to protection effect from the attack of free arsenic species. Likewise, the patients with low-proteinemia besides liver and/or renal dysfunctions might frequently develop arsenic-mediated side effects. Understandably, further investigation of the detailed information about these proteins is needed.

In conclusion, we clarified the arsenic speciation for the first time in BM plasma, and found that speciation profiles of BM plasma were very similar to those of PB plasma. We also demonstrated that the total arsenic concentrations of HMW-F were much higher in BM plasma than those in PB plasma. These results may further provide not only significance of clinical application of ATO, but also a new insight into host defense mechanisms in APL patients undergoing ATO treatment. Of note, it has been demonstrated that targeting for the PML with ATO could lead to loss of self-renewal capability of LSCs, which is closely linked to chemotherapy resistance and disease relapse, in CML [18], suggesting that ATO would be a promising candidate for a LSCs-targeted therapy [17]. Our results thus may provide new evidence for LSCs-targeted therapy in APL patients based on the following facts: 1) PML-RARα can be detected in more than 95% of APL patients [2], 2) long-term follow-up of newly diagnosed patients with APL treated with single ATO therapy shows a high 5-year disease-free survival (DFS) rate and overall survival (OS) rate [35-37]. It is well known that the blood trough levels correlated with clinical benefit better than either maximum levels or area under the curve (AUC). Similar to our and other group's previous reports [13,38], the plasma trough levels of As(III) were much lower compared to a previous report [6]. These results thus leave open a possibility that much lower concentrations of As(III) possess biological effects such as differentiation-inducing activity, although we could not exclude whether other arsenicals, such as As(V) and methylated arsenic metabolites, alone or their combination contribute to the effects based on a previous report showing that methylated arsenic metabolites also possess cytocidal activity against leukemia and lymphoma cells, consequently may contribute to the therapeutic effect of ATO in APL patients [39]. Details of the study are currently underway.

## Competing interests

The authors declare that they have no competing interests.

## Authors' contributions

NI and YY contributed equally to this study; NI, YY, and Y(Yukio)H performed experiments; BY, NI, and YY analyzed results and presented; AH and Y(Yoshihiro)H assisted interpretation of the result with BY, NY, and YY; BY, NI and JT designed the research and wrote the manuscript; HT directed and oversight the project. All authors read and approved the final manuscript.
